# Organic and Inorganic Amendments Shape Bacterial Indicator Communities That Can, In Turn, Promote Rice Yield

**DOI:** 10.3390/microorganisms10020482

**Published:** 2022-02-21

**Authors:** Chongwen Qiu, Yuanyuan Bao, Evangelos Petropoulos, Yiming Wang, Zhenfang Zhong, Yaozhi Jiang, Xuhong Ye, Xiangui Lin, Youzhi Feng

**Affiliations:** 1 Ministry of Agriculture Key Laboratory for Northeast Preservation of Cultivated Land, National Engineering Research Centre for Efficient Utilization of Soil and Fertilizer Resources, College of Land and Environment, Shenyang Agricultural University, Shenyang 110065, China; qcwabc@163.com; 2 Guangdong Haina Institute of Agriculture, Huizhou 516000, China; hnnyzzf@126.com (Z.Z.); 18871996518@163.com (Y.J.); 3 State Key Laboratory of Soil and Sustainable Agriculture, Institute of Soil Science, Chinese Academy of Sciences, Nanjing 210008, China; yybao@issas.ac.cn (Y.B.); ymwang@issas.ac.cn (Y.W.); yzfeng@issas.ac.cn (Y.F.); 4 School of Civil Engineering and Geosciences, Newcastle University, Newcastle upon Tyne NE1 7RU, UK; vagpetrop@gmail.com

**Keywords:** bacterial community, fertilization, indicator species, rice yield, soil chemical properties, soil microbial properties

## Abstract

The dynamic patterns of the belowground microbial communities and their corresponding metabolic functions, when exposed to various environmental disturbances, are important for the understanding and development of sustainable agricultural systems. In this study, a two-year field experiment with soils subjected to: chemical fertilization (F), mushroom residues (MR), combined application of chemical fertilizers and mushroom residues (MRF), and no-fertilization (CK) was conducted to evaluate the effect of fertilization on the soil bacterial taxonomic and functional compositions as well as on the rice yield. The highest rice yield was obtained under MRF. Soil microbial properties (microbial biomass carbon (MBC), microbial biomass nitrogen (MBN), urease, invertase, acid phosphatase, and soil dehydrogenase activities) reflected the rice yield better than soil chemical characteristics (soil organic matter (SOM), total N (TN), total K (TK), available P (AP), available K (AK), and pH). Although the dominant bacterial phyla were not significantly different among fertilizations, 10 bacterial indicator taxa that mainly belonged to Actinobacteria (*Nocardioides*, *Marmoricola*, *Tetrasphaera*, and unclassified *Intrasporangiaceae*) with functions of xenobiotic biodegradation and metabolism and amino acid and nucleotide metabolism were found to strongly respond to MRF. Random Forest (RF) modeling further revealed that these 10 bacterial indicator taxa act as drivers for soil dehydrogenase, acid phosphatase, pH, TK, and C/N cycling, which directly and/or indirectly determine the rice yield. Our study demonstrated the explicit links between bacterial indicator communities, community function, soil nutrient cycling, and crop yield under organic and inorganic amendments, and highlighted the advantages of the combined chemical and organic fertilization in agroecosystems.

## 1. Introduction

Modern agriculture is facing the challenge of meeting the increasing demand for agricultural products [1]. Chemical fertilization is considered an effective way to maintain the agricultural yield in agroecosystems [2]; however, excessive use of chemical fertilizers has triggered a series of environmental problems (accumulation of heavy metals in soil, water eutrophication, and soil acidification) [3,4,5]. Organic fertilization, such as with animal manure and/or plant residues, is an environmentally friendly way to improve the soil nutrition and increase the agricultural yield [6]. This strategy though results in a lower degree of soil nutrition than that from chemical fertilizers [7]. Simultaneous application of chemical and organic fertilizers is considered to be an ideal strategy that benefits the soil state, crop yield, and agricultural sustainability.

Microorganisms, especially the relevant functional species, play vital roles in agricultural soil multi-nutrient cycling and crop disease biocontrol [8,9]. Such taxa not only use the substances from the fertilizers to form easily absorbed nutrients for the plants but also indirectly regulate soil pH through biochemical metabolism, which provides the optimum conditions for microbial community growth and crop yield promotion [10]. Generally, fertilization strategies tend to influence the soil microbial community structure [11], which can, in turn, influence the microbial ecological functions [12,13]. Many studies have evaluated the effect of fertilization on the soil microbial communities and have assessed how they both promote the crop yield [1,14]. Exploring the microbial community functions, rather than just identifying the microbial composition, is considered essential to understanding the actual microbial functional roles in a given ecosystem [15,16]. Unfortunately, the effect of simultaneous chemical-organic fertilization on community functions is a topic that remains unexplored.

Moreover, on numerous occasions, the entire microbial community’s composition has been found, at the taxonomic level, to have demonstrated only minor and often insignificant changes, with only several indicative species significantly differing, when subjected to various fertilization amendments [17,18]. This represents a significant scientific gap in research on the properties of fertilization and how these link the microbial indicator communities and their metabolic functions with crop yield. Understanding the functions of specific microbial indicator species is challenging as it requires multidisciplinary research involving microbial ecology, microbiology, and ecosystem function analysis. Thus, studies focusing on the link between the crop yield and microbial indicator taxa, including their relevant functions, in the presence of simultaneous organic and inorganic amendments to fertilization are rare.

In this study, Random Forest modeling and function prediction, combined with statistical analysis, were used to study the effect of fertilization on the rice yield and taxonomic and functional compositions of the indicator microorganisms. Since most of the rice cultivation period during experimentation was under anoxic conditions, the activities of fungi were expected to be limited [19]; thus, in this study, there was only a focus on the bacterial kingdom. We hypothesized that inorganic and organic amendments shape the bacterial indicator communities, with the latter serving an essential function in promoting the rice yield. To test this hypothesis (by evaluating the effect of organic-inorganic fertilization on crop yields and microbial communities and functions), a two-year field fertilization experiment was conducted on a rice-rice cropping system.

## 2. Methods

### 2.1. Field Site and Experiment Description

The experiment was conducted at a long-term crop trial site in Huizhou, Guangdong, China (23°15′ N, 114°49′ E), during rice-rice (“Meixiangzhan 2”) rotation at the beginning of March 2019. The local climate is subtropical monsoon with annual precipitation of ~2000 mm and an average annual temperature of 22.8 °C. Four fertilization treatments were prepared: CK (without fertilization), F (mineral NPK fertilizers), MR (mushroom residues), and MRF (mineral NPK fertilizers plus mushroom residues). The mineral NPK fertilizers were obtained from Hubei Sinochem & Orient Fertilizer Co., Ltd. (Sinochem, Wuhan, China). The N content of mineral NPK fertilizers was 46%. The mushroom (*Flammulina velutipes*) residues were obtained from Guangdong Xuerong Bio-Technology Co., Ltd. (Xuerong, Huizhou, China). The main properties of mushroom residues were 1.84% total N, 0.70% total P, 1.25% total K, and 63.80% organic matter. The F, MR, and MRF treatments were designed to supply the same rate of total N (i.e., 135 Kg N ha^−1^). For the MRF treatment, i) the mineral NPK fertilizer and ii) the mushroom residues used contained 50% of the total N. For each treatment, eight replicates were randomly conducted.

### 2.2. Soil Sampling and Analysis

Paddy soils exposed to a two-year application of different fertilization treatments were collected from the plow layer (0–20 cm) of each replicate plot of each treatment using a 30-mm-diameter auger; sampling took place after the harvesting of rice. The soil sample from each replicate was a combination of five soil cores that were randomly collected, and a total of 32 samples were prepared for analysis in this study. Each sample was sieved through a 2-mm mesh and then screened and divided into two subsamples: i) air-dried for soil chemical and enzyme activity analyses, and ii) stored at −80 °C for DNA extraction and molecular analysis.

The soil chemical properties were determined according to the protocols of Lu [20]. The soil organic matter (SOM) was determined using a volumetric K_2_Cr_2_O_7_-heating method. The soil total N (TN) was determined by Kjeldahl digestion. The soil total K (TK) was digested by hydrofluoric acid (HF)-perchloric acid (HClO_4_) and determined by molybdenum-blue colorimetry and flame photometry. The soil available P (AP) was determined using the molybdenum-blue method. The soil available K (AK) was extracted by ammonium acetate and determined by flame photometry. The soil pH was determined from soil-water suspensions (1:2.5 *v/v*) using a pH meter.

### 2.3. Soil Enzyme Activities and Microbial Biomass Analysis

The soil urease (EC 3.5.1.5) activity was determined with the indophenol blue colorimetric method [21]. The soil invertase (EC 3.2.1.26) activity was determined by the 3, 5-dinitrosalicylic acid method [22]. The soil acid phosphatase (EC 3.1.3.2) activity was assayed by Hoffman’s method [23]. The soil dehydrogenase activity was estimated by the reduction of 2, 3, 5-triphenyltetrazolium chloride (TTC) to 2, 3, 5-triphenylformazan (TPF) [24].

The microbial biomass carbon (MBC) and nitrogen (MBN) were determined according to the validated methods described by Vance et al. [25]. In brief, fresh soils were firstly fumigated for 24 h, and then the soluble carbon and nitrogen were extracted using 0.5 mol L^−1^ K_2_SO_4_ for 60 min on a rotary shaker. MBC and MBN were then calculated as described by Joergensen [26] and Joergensen and Mueller [27], respectively.

### 2.4. DNA Extraction, PCR Amplification, and MiSeq Sequencing

We extracted 0.5 g soil from each sample using a FastDNA^®^ SPIN Kit for Soil (MP Biomedicals, Santa Ana, CA, USA). The extracted DNA was quantified using a Nanodrop 2000 (ThermoFisher, Wilmington, NC, USA) and then stored at −20 °C for further use/analysis. The 519F/907R primer set was used to amplify the bacterial 16S rRNA gene V4–V5 fragments according to Feng et al. [28]. Then, the TruSeq™ DNA Sample Prep LT Kit (Illumina Inc., CA, USA) was used to normalize all the purified PCR products in equimolar amounts. Following this, the libraries were sequenced with the Illumina MiSeq sequencing platform (Illumina Inc., CA, USA).

### 2.5. Processing High-Throughput Sequencing Data

Amplicon libraries were processed using the Quantitative Insights Into Microbial Ecology (QIIME 1) (v.1.9.1, USA) pipeline [29]. Sequences with a quality score below 25 and a length smaller than 300 bp were trimmed. Then, the quality reads were divided into operational taxonomic units (OTUs) using the 97% identity threshold, and the most abundant sequence of each OTU was chosen as the representative sequences. Taxonomy information was assigned to each OTU concerning a subset of the Greengenes database. In total, 530,266 quality bacterial 16S rRNA gene V4–V5 fragment sequences were obtained (12,222~21,682 sequences per sample), with a median value of 15,913 sequences per sample.

### 2.6. Bacterial Community Function Predictions

Bacterial community functions under different fertilization treatments were analyzed by PICRUSt [30]. Firstly, the normalized OTU table was submitted to PICRUSt v.1.1.2 for metagenome prediction, and then the final metagenomic functions based on KEGG (Kyoto Encyclopedia of Genes and Genomes) pathways were created.

### 2.7. Random Forest Model

The relative levels of abundance of the bacterial taxa against the different fertilization treatments were regressed at the genus level using the default parameters of R (implementation of the algorithm in the R package included “Random Forest”, ntree = 1000) [31]. Then 10-fold cross-validation was used to identify the number of marker taxa. The RF model was further applied to estimate the importance of the bacterial indicator taxa according to the degree to which they explained the soil multi-nutrient cycling [32]. A multiple regression model was used to validate the outcome of RF with the lm and calc.relimp function of the “relaimpo” package. Then, the RF model was further performed to evaluate the important bacterial metabolic functions under each fertilization treatment by regressing the relative levels of abundance of the predicted functional genes against fertilization treatments.

### 2.8. Statistical Analysis

In this study, all samples were randomly rarified to 12,222 sequences for downstream analyses since an even depth of sampling is required for alpha (α) and beta (β) diversity comparisons [33]. The bacterial community distribution patterns from different fertilization treatments were further displayed by nonmetric multidimensional scaling analyses (NMDS) based on the Bray-Curtis distance. Permutational multivariate analysis of variation (PERMANOVA) tests [34] were conducted to test the statistically significant differences of the Bray-Curtis-based community compositions among different fertilization treatments (using vegan package in R software) (v.2.2-1). To show the relationship between the rice yield and soil chemical and biological properties, polynomial regression curves were plotted. The soil chemical and biological properties were calculated using the combined Z-scores of SOM, TN, TK, AP, AK, and pH, and the MBC, MBN, soil urease activity, soil invertase activity, soil acid phosphatase activity, and soil dehydrogenase activity, respectively. The Procrustes test was used to determine the links between the bacterial community composition and community function [35]. Statistical procedures were calculated using IBM Statistical Product and Service Solutions (SPSS) Statistics for Windows (v.13, Armonk, NY, USA). Mean separation among decomposition stages was evaluated by one-way ANOVA followed by posthoc Tukey’s HSD tests. A difference of *p* < 0.05 was considered statistically significant.

## 3. Results

### 3.1. Crop Yield, Soil Chemical and Biological Properties under Different Fertilization Regimes

**Figure 1:**
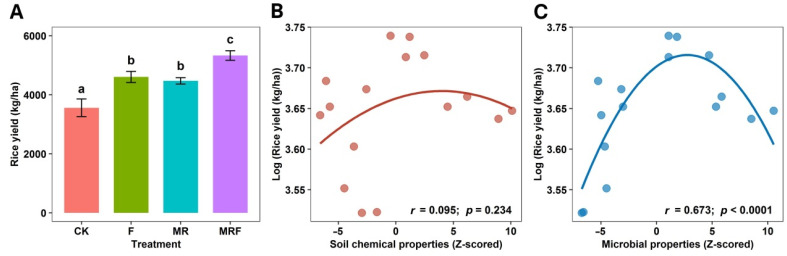
Crop yield under different fertilization treatments (**A**) and the quadratic correlations of the rice yield with soil chemical (**B**) and microbial (**C**) properties. Soil chemical properties were calculated using the combined Z-scores of SOM, TN, TK, AP, AK, and pH, while soil microbial properties were calculated using the combined Z-scores of MBC, MBN, soil urease activity, soil invertase activity, soil acid phosphatase activity, and soil dehydrogenase activity. CK: without fertilization, F: mineral NPK fertilizers, MR: mushroom residues, MRF: mineral NPK fertilizers plus mushroom residues. The error bars indicate standard deviations of means. Different letters over error bars denote significant differences (*p* < 0.05).

Both the soil’s chemical and biological properties were altered by different fertilization regimes (Supplementary Table S1). Most of the soil properties’ indices, such as TN, AP, AK, SOM, MBC, MBN, acid phosphatase, and dehydrogenase, reached their peak values when subjected to MR (Supplementary Table S1). The rice yields from different fertilization regimes are presented in Figure 1A. Overall, fertilization significantly increased the rice yield (*p* < 0.05). The maximum yield was observed under MRF, and no significant difference between F and MR treatments in terms of rice yield (*p* > 0.05) was observed. Many of the biological properties, such as soil urease, invertase, acid phosphatase, and dehydrogenase, were significantly correlated (*p* < 0.05, Mantel test) with the soil bacterial community composition and the corresponding microbial activities, an observation that is also well-supported in the literature [36,37,38,39]. Thus, biological properties can be referred to as “microbial properties”. It was also found that soil microbial properties had a strong, significant correlation (*r* = 0.673, *p* < 0.0001) with the rice yield (Figure 1C); however, no significant correlation between soil chemical properties and the rice yield was observed (*r* = 0.095, *p* = 0.234) (Figure 1B).

### 3.2. Taxonomic Distribution of the Bacterial Communities under Different Fertilization Regimes

**Figure 2:**
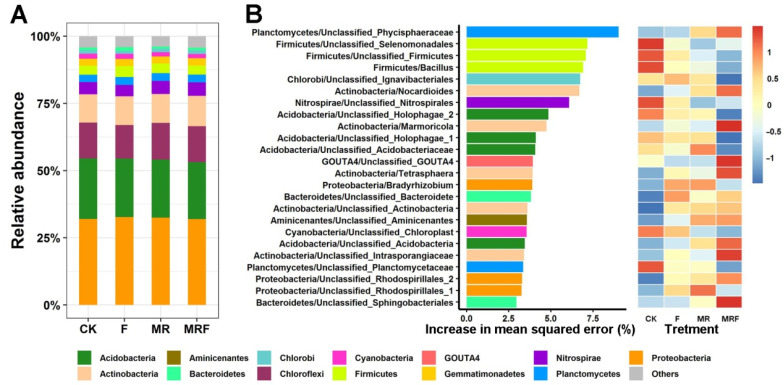
Phylum-level compositions of the bacterial community under different fertilization treatments (**A**). Predictor importance (percentages of increase in mean square error) of the top 24 bacterial taxonomic biomarkers and their relative levels of abundance (standardized by Z-score transformation) under each fertilization treatment (**B**). CK: without fertilization, F: mineral NPK fertilizers, MR: mushroom residues, MRF: mineral NPK fertilizers plus mushroom residues.

Bacterial communities with an average relative abundance of over 1% are shown in Figure 2A (Proteobacteria (32.3%), Acidobacteria (21.8%), Chloroflexi (13.2%), Actinobacteria (10.8%), Nitrospirae (4.7%), Firmicutes (3.6%), Planctomycetes (2.8%), Gemmatimonadetes (2.7%), Cyanobacteria (1.7%), Chlorobi (1.2%), and Bacteroidetes (1.1%)). The patterns of the relative abundance of the dominant phyla (average relative levels of abundance > 10%) among different fertilization treatments show that Proteobacteria, Acidobacteria, and Chloroflexi did not significantly differ among different treatments (*p* > 0.05, Supplementary Figure S1), with the exception being the abundance of Actinobacteria (*p* = 0.036, Supplementary Figure S1). The important bacterial genera, defined as biomarker taxa for each fertilization, were further examined by the RF model (Figure 2B). The RF model explained 57.64% of the bacterial variance related to different fertilization regimes. Twenty-four important genera were identified and used as representative biomarker taxa based on the minimum cross-validation error obtained (Supplementary Figure S2). Generally, 10 bacterial indicator taxa (unclassified Phycisphaeraceae, *Nocardioides*, *Marmoricola*, unclassified GOUTA4, *Tetrasphaera*, unclassified Actinobacteria, unclassified Acidobacteria, unclassified Intrasporangiaceae, unclassified Rhodospirillales, and unclassified Sphingobacteriales) distinctly responded (these bacterial taxa were referred to as “indicator bacterial taxa”) to the MRF treatment.

### 3.3. Variation in Bacterial Community Composition among Different Fertilization Regimes

**Figure 3:**
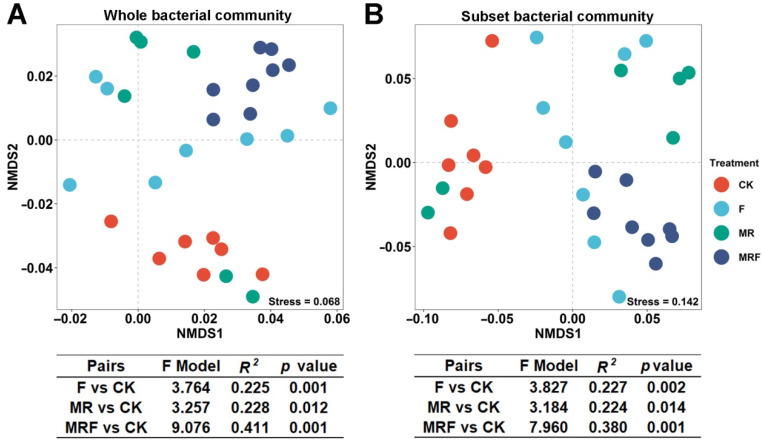
Nonmetric multidimensional (NMDS) and PERMANOVA analyses of the whole (**A**) and subset (by filtering out the ten bacterial indicator taxa found in MRF from the whole bacterial community) (**B**) bacterial communities under different fertilization treatments based on Bray-Curtis distance. CK: without fertilization, F: mineral NPK fertilizers, MR: mushroom residues, MRF: mineral NPK fertilizers plus mushroom residues.

The entire bacterial community composition, among different fertilization treatments, was further visualized by an NMDS plot based on the Bray-Curtis distance (Figure 3A). This showed that the bacterial communities were separated into four clusters. Significant differences between the bacterial communities based on the fertilization treatments (F, MR, and MRF) and the CK were confirmed by a PERMANOVA pairwise test (*p* < 0.05, Figure 2A). In addition, the F score between MRF and CK (F score = 9.076) was higher than that for MR vs. CK (F score = 3.257) or F vs. CK (F score = 3.764). This suggests that the extent of the bacterial community changes under MRF is larger than that under F and MR. To understand the roles of the previously mentioned top-10 bacterial indicator taxa in the community under MRF (Figure 2B), we visualized the subcommunity (community excluding the 10 key taxa from the whole bacteria community) composition among different fertilization treatments (Figure 3B). Although significant differences in the bacterial subcommunity composition were found among different fertilization treatments, the difference between MRF and CK was found to be less significant (with a lower F score = 7.960, Figure 3B) than that in entire community comparisons (with a higher F score = 9.076, Figure 3A).

### 3.4. Dynamic Change in Compositions of Bacterial Metabolic Functions under Different Fertilizations

**Figure 4:**
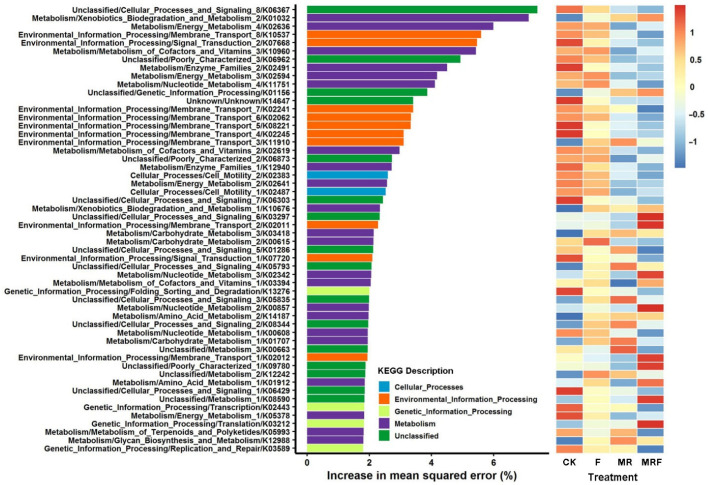
Predictor importance (percentages of increase in mean square error) of the top-54 bacterial metabolism functions under each fertilization treatment. CK: without fertilization, F: mineral NPK fertilizers, MR: mushroom residues, MRF: mineral NPK fertilizers plus mushroom residues.

The RF model was further performed to evaluate the important bacterial metabolic functions under each fertilization treatment (Figure 4). The RF model explained 66.63% of the variance in the bacterial metabolic functions related to different fertilizations. The minimum cross-validation error was obtained from the 54 metabolic functions that were chosen (Supplementary Figure S3), and these were used as the representative metabolic functions under different fertilization treatments. More specifically, the 13 bacterial indicators of KEGG Orthology functional genes that got enriched in MRF treatment were K03212, K08590, K01912, K09780, K02012, K00857, K03394, K02342, K02011, K03297, K01156, K10676, and K01032, with the corresponding functions of translation, membrane transport, xenobiotic biodegradation and metabolism, and metabolism of amino acids, nucleotide, cofactors, and vitamins (Figure 4).

### 3.5. Relationship between Bacterial Indicator Community, Community Function, Soil Nutrient Cycling, and Crop Yield

**Figure 5:**
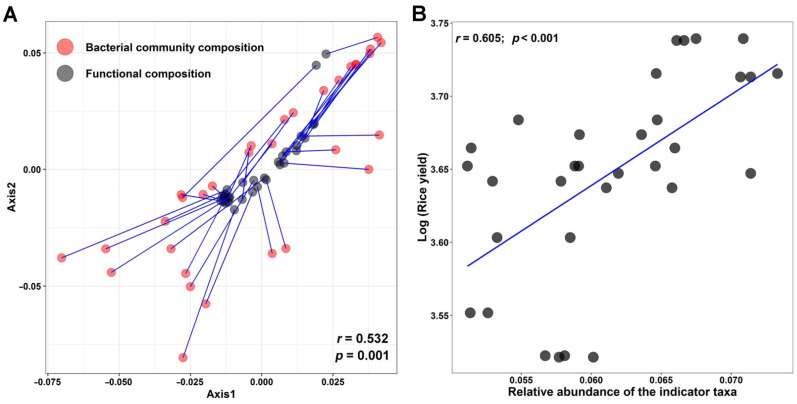
Relationship between the bacterial indicator community (i.e., 10 bacterial indicator taxa enriched in MRF treatment) and indicator function (i.e., 13 bacterial indicators of KEGG Orthology functional genes enriched in MRF treatment) found in MRF treatment (**A**). Linear correlation of crop yields with the relative abundance of the bacterial indicator taxa enriched in MRF (**B**). CK: without fertilization, F: mineral NPK fertilizers, MR: mushroom residues, MRF: mineral NPK fertilizers plus mushroom residues.

The Procrustes test was used to reveal the relationship between the bacterial indicator communities (Figure 3) and the metabolic functions (Figure 4) observed in MRF treatment (Figure 5A). It was found that the indicator bacterial community composition in MRF was significantly correlated with the indicators of metabolic functions (*r* = 0.532, *p* = 0.001). The RF model was further used to estimate the importance of the bacterial indicator taxa enriched in each treatment for explaining soil multi-nutrient cycling. Specifically, it was found that 10 bacterial indicator taxa enriched in the MRF treatment were main drivers for soil dehydrogenase (40.0%), acid phosphatase (32.3%), pH (27.5%), TK (25.7%), and C/N (15.4%) cycling (Supplementary Table S2). In addition, the linear regression model showed that the relative abundance of the bacterial indicator taxa was significantly correlated with the rice yield (*r* = 0.605, *p* < 0.001) (Figure 5B).

## 4. Discussion

### 4.1. Microbial Properties of the Soil Community Reflect Crop Yield Better Than Soil Chemical Characteristics

In this study, the rice yield and the soil properties under the four fertilization treatments were evaluated. The highest rice yield was obtained under the MRF treatment. This observation is consistent with that of Mi et al. [40] who also found that the rice grain yields under NPK-cattle manure and NPK-rice straw amendments were significantly higher than when under NPK or CK alone. This yield promotion may be attributed to the complexity of fertilization, which influences/changes the soil properties, and thus, improves the crop yield [40]. This plausible scenario is consistent with the significantly different chemical and microbial properties of the soils when subjected to different fertilization regimes (Supplementary Table S1). This observation is aligned with Zhao et al. [17], Ye et al. [41], and Lu et al. [14], who found that soil chemical (e.g.,; pH, TN, AN, AP, and AK) and microbial properties (MBC, MBN, urease activity, urease activity, and alkaline phosphatase) significantly differ when subjected to complex fertilization.

More interestingly, although the MR treatment achieved the highest soil chemical and microbial indices (Supplementary Table S1), the highest rice yield was obtained under the MRF treatment. This outcome suggests that there was a nonlinear relationship between the rice yield and soil properties, which would be consistent with the quadratic correlations between soil properties (especially for soil microbial properties) and the rice yield (Figure 1B,C). This observation is consistent with previous work related to the impact of soil properties on the crop yield [42,43]; these studies highlighted that it is the optimal, rather than maximum, soil chemical and microbial values that benefit crop productivity. There could be various factors explaining this phenomenon, for example, higher and/or lower-than-optimum soil pH is usually considered a major obstacle to crop productivity [42]. In addition, the combined effect of soil properties (e.g.,; pH and/or nitrogen) on plant growth may be superimposed. For example, it was found that high soil pH and nitrogen could superimpose on each other and together have a negative effect on the rice yield [42]. Another potential explanation may be that there could have been an optimal enzyme concentration that accelerated the hydrolysis and fermentation of the available organic carbon form for plant growth. An increase in enzyme concentration can indeed accelerate plant growth due to a surplus in carbon availability; however, a continuous increase in enzyme concentration may result in no marked effect on plant production [44]. In this study, the soil chemical and microbial properties may have become optimal for rice plant growth under MRF. Collectively, the abovementioned factors provide plausible reasons for why MRF, with its moderate soil chemical and microbial indices, achieved the maximum rice yield.

Moreover, the greater correlation between soil microbial properties and the rice yield (Figure 1C) compared to the one between soil chemical properties and the rice yield (Figure 1B) suggests that soil microbial properties are more sensitive to the fertilization type. Subsequently, microbial properties can stand as a potential indicator for assessing the rice yield. Our result was consistent with the study of Wu et al. [1] who found that although both soil biotic (e.g., microbes) and abiotic factors (e.g., nutrient elements) have a critical effect on crop yield, biological properties reflect the actual conditions of crop growth better than soil physicochemical characteristics [1]. There could be a couple of justifications for that. Specifically, the flow of matter and energy in the soil matrix have been proven to have a great influence on crop yields [45,46]. Microorganisms can secrete many of the enzymes needed for soil nutrition and energy conversion [1,47], and as such, can act as a bridge between plant roots and the environment they inhabit, so the plant can achieve high crop yields. This is a phenomenon related to the ability of both microbes and plants to coexist and act synergistically [48,49]. Another potential justification may be that soil chemical characteristics, such as SOM, AK, and pH, can influence soil microbial community composition, which can, in turn, indirectly affect the crop yield [14]. The abovementioned phenomena collectively imply that the high rice yield achieved under MRF may have resulted from specific microbial communities enriched by the specific fertilization type. This, in turn, led to optimal soil biochemical conditions for both bacteria and rice crop growth.

### 4.2. Organic and Inorganic Amendments Indirectly Affect Crop Yield by Shaping the Bacterial Indicator Communities

In numerous soil microbial studies, the dominant members of the communities were over-focused as they were generally considered more active and important than other taxa participating in biogeochemical cycling [50]. However, many dominant species in soils have been proven not to necessarily respond to environmental changes [18]. As such, they may dilute the effect of environmental disturbances on some that are maybe less dominant in the community species; the latter may relate with more essential functions for plant growth. By revealing these microbial indicator taxa and the corresponding functions, we can theoretically clarify the response of the former to different environmental changes. However, to the best of our knowledge, such information is still unknown as this field is yet unexplored.

In this study, although the dominant bacterial phyla were not significantly different among fertilization treatments (Figure 2A and Supplementary Figure S1), different bacterial indicator taxa were found to be enriched under specific fertilization regimes (Figure 2B). This observation is consistent with the findings of Zhao et al. [17] and Yao et al. [18] who also found that the majority of the dominant microbial species were considered non-responsive, and only a subset of more sensitive species responded differently to environmental disturbances. In this study, only 10 bacterial indicator taxa were found to strongly respond to MRF treatment. The difference in bacterial community compositions between MRF and CK decreased when these 10 bacterial indicator taxa were screened out of the entire community (Figure 3). This result implies that it is these 10 bacterial indicator taxa that altered the original soil bacterial community composition after the MRF amendment. Thus, we speculate that these 10 bacterial indicator taxa are those playing vital roles in affecting the rice yield due to the specific functions that they possess.

As mentioned earlier, the RF model was used to further reveal the bacterial indicator functions under different fertilizations. Interestingly, it was found that 13 bacterial indicators of KEGG Orthology functional genes, such as K01032 and K10676, responsible for xenobiotic biodegradation were enriched in the MRF treatment (Figure 4). The strong Procrustes correlation (*r* = 0.532, *p* = 0.001, Figure 5A) between the bacterial indicator community composition and the indicator organisms’ metabolism functions in MRF suggests that the enriched KEGG Orthology functions may be mainly derived from the enriched bacterial indicator taxa. This suggestion is consistent with the functional analysis of the bacterial phylum of Actinobacteria, which accounted for 50% of the total indicator species in MRF (Figure 2B). For example, the relative levels of abundance of actinobacterial functional genes, responsible for xenobiotic biodegradation and metabolism including carbohydrate metabolism, were significantly higher in MRF than in CK, NPK, and MR treatments (*p* < 0.05). Mushroom residues contain poly-aromatic hydrocarbons (a kind of organic matter derived from mushroom residues) and are considered xenobiotic compounds [51], or in other words, a substrate that can specifically select for Actinobacteria to degrade and convert to nutrients for the rice plant to uptake. This observation is in line with previous studies indicating that Actinobacteria play a key ecophysiological role in plant residue decomposition [52,53].

The RF model further confirmed that the 10 bacterial indicator taxa enriched in MRF treatment have specific functions and act as drivers for many of the soil multi-nutrient cycling parameters (such as soil dehydrogenase, acid phosphatase, pH, TK, and C/N) (Supplementary Table S2). Hence, such indicator taxa and their functions directly determine the plant yield [42,54,55,56,57]. In addition, microbial-driven soil chemical nutrient cycling such as soil C/N and pH can significantly influence soil hydrolase activities, which indirectly affect the plant yield [58]. Overall, the significant correlations between the relative levels of abundance of the 10 bacterial indicator taxa and the rice yield (Figure 5B), combined with the abovementioned results, collectively confirm our hypothesis that organic and inorganic amendments shape the bacterial indicator communities to serve essential functions that promote the rice yield.

## 5. Conclusions

Different from previous relevant studies, which usually only focused on the microbial communities, the present study focused on the links between the rice yield and bacterial indicator taxa and their functions under different fertilization regimes. Our results indicate that soil microbial properties reflect the rice yield better than soil chemical characteristics in crops subjected to different fertilizations. Only several bacterial indicator taxa—for example, Actinobacteria—rather than the entire bacterial community, are specifically enriched under organic and inorganic amendments. These indicator bacterial taxa possess specific functions such as xenobiotic biodegradation and carbohydrate metabolism that act as drivers for soil multi-nutrient cycling, promoting the rice yield. Combining the taxonomic and functional information on the microbial indicator species under different fertilization regimes can provide an overall understanding of the mechanisms that improve crop productivity by organic and inorganic amendments.

## Data Availability

The sequences were deposited into the DDBJ database (accession no. DRA012207).

## Supplementary Materials

The following supporting information can be downloaded at: https://www.mdpi.com/article/10.3390/microorganisms10020482/s1, Figure S1: Relative abundance levels of dominant bacteria among different fertilization regimes, Figure S2: The number of bacterial genera against the cross-validation error curve, Figure S3: The number of metabolism functions against the cross-validation error curve, Table S1: Soil chemical and microbial properties under different fertilization regimes, Table S2: Random Forest (RF) mean predictor importance (percentage of increase of mean square error) of the bacterial indicator taxa enriched in each treatment as drivers for the soil multi-nutrient cycling index (i.e., SOM, TN, TK, AP, AK, pH, C/N, MBC, MBN, urease activity, invertase activity, acid phosphatase activity, and dehydrogenase activity).
