# Supplementary material for: Organic and Inorganic Amendments Shape Bacterial Indicator Communities That Can, In Turn, Promote Rice Yield
Source: Microorganisms. 2022 Feb 21;10(2):482. doi: 10.3390/microorganisms10020482 (PMC8880095; doi:10.3390/microorganisms10020482)
Supplement: Supplementary file 1 [file microorganisms-10-00482-s001.zip › microorganisms-1556424-supplementary.pdf]

Article

Organic and Inorganic Amendments Shape Bacterial Indicator Communities That Can, In Turn, Promote Rice Yield

Chongwen Qiu [1,2,†], Yuanyuan Bao [3,†], Evangelos Petropoulos [4], Yiming Wang [3], Zhenfang Zhong [2], Yaozhi Jiang [2], Xuhong Ye [1,*], Xiangui Lin [3,*] and Youzhi Feng [3]

[1] Ministry of Agriculture Key Laboratory for Northeast Preservation of Cultivated Land, National Engineering Research Centre for Efficient Utilization of Soil and Fertilizer Resources, College of Land and Environment, Shenyang Agricultural University, Shenyang 110000, China; qcwabc@163.com (C.Q.); yexuhong1980@163.com (X.Y.)

[2] Guangdong Haina Institute of Agriculture, Huizhou 516000, China; hnnyzzf@126.com (Z.Z.); 18871996518@163.com (Y.J.)

[3] State Key Laboratory of Soil and Sustainable Agriculture, Institute of Soil Science, Chinese Academy of Sciences, Nanjing 210008, China; yybao@issas.ac.cn (Y.B.); ymwang@issas.ac.cn (Y.W.); xglin@issas.ac.cn (X.L.); yzfeng@issas.ac.cn (Y.F.)

[4] School of Civil Engineering and Geosciences, Newcastle University, Newcastle upon Tyne NE1 7RU, UK; vagpetrop@gmail.com

* Correspondence: yexuhong1980@163.com (X.Y.); xglin@issas.ac.cn (X.L.); Tel.: +086-024-82887155 (X.Y.); +086-025-86881589 (X.L.); Fax: +086-024-82887155 (X.Y.); +086-025-86881000 (X.L)

† These authors have contributed equally to this work.

Supplementary Materials.

Table S1. Soil chemical and microbial properties under different fertilization regimes.

Treatment	Soil chemical properties							Soil microbial properties					
	pH	TN	AP	TK	AK	SOM	C/N	MC	MN	Urease	Acid phosphatase	Dehydrogenase	Invertase
	1:2.5	g kg^{-1}	mg kg^{-1}	%	mg kg^{-1}	g kg^{-1}	/	mg kg^{-1}	mg kg^{-1}	mg NH_4^+-N g^{-1} *d.w.s* day^{-1}	mg hydroxybenzene g^{-1} *d.w.s* day^{-1}	mg TPF g^{-1} *d.w.s* $hour^{-1}$	mg glucose g^{-1} *d.w.s* day^{-1}
CK	5.14 ± 0.05b	1.44±0.03a	23.28 ± 0.87a	2.06 ± 0.06b	69.51 ± 6.31a	24.23 ± 0.9a	9.04 ± 0.16a	496.02 ± 21.27a	97.21 ± 2.95a	0.48 ± 0.03a	1.72 ± 0.07a	2.73 ± 0.08a	21.78 ± 0.56b
F	4.98 ± 0.06a	1.44 ± 0.13a	28.49 ± 1.71b	1.94 ± 0.09a	57.71 ± 5.25a	25.65 ± 0.32a	9.66 ± 0.96a	489.26 ± 28.05a	104.44 ± 4.05a	0.55 ± 0.01a	2.14 ± 0.14b	3.02 ± 0.13b	20.5 ± 0.65a
MR	5.23 ± 0.08c	2.12 ± 0.13c	42.11 ± 3.64c	2.22 ± 0.07c	89.66 ± 12.14b	36.07 ± 2.21c	9.13 ± 0.1a	634.17 ± 61.66c	142.88 ± 14c	0.72 ± 0.04b	2.97 ± 0.09c	3.9 ± 0.03d	23.83 ± 0.74c
MRF	5.21 ± 0.05bc	1.69 ± 0.06b	30.56 ± 0.7b	2.25 ± 0.03c	59.27 ± 10.26a	29.05 ± 1.47b	9.23 ± 0.29a	560.1 ± 9.81b	120.43 ± 7.63b	0.69 ± 0.13b	2.23 ± 0.05b	3.42 ± 0.06c	23.58 ± 0.22c

Different letters denote significant differences ($P < 0.05$).

Table S2. Random forest (RF) mean predictor importance (percentage of increase of mean square error) of the bacterial indicator taxa enriched in each treatment as drivers for the soil multi-nutrient cycling index (that is, SOM, TN, TK, AP, AK, pH, C/N, MBC, MBN, urease activity, invertase activity, acid phosphatase activity, and dehydrogenase activity).

Treatment	Bacterial taxa	Increase in mean square error (MSE) (%)												
		Soil chemical properties							Soil enzyme activities				Microbial biomass	
		SOM	TN	TK	AP	AK	pH	C/N	Urease activity	Phosphatase activity	Dehydrogenase activity	Invertase activity	Microbial biomass C	Microbial biomass N
CK	Unclassified_Selenomonadales	5.5	3.1	1.0	6.3	1.3	0.9	3.6	5.9	7.7	7.4	2.1	2.3	7.6
	Unclassified_Firmicutes	4.3	5.3	6.7	5.8	4.2	7.4	3.1	3.5	4.0	6.1	9.5	6.7	4.9
	Bacillus	1.9	3.8	3.7	3.8	0.7	4.6	3.2	1.9	2.0	2.3	5.0	4.0	0.9
	Unclassified_Nitrospirales	9.2	8.4	0.3	6.4	4.6	2.8	1.9	7.8	7.9	12.6	5.0	6.5	9.6
	Unclassified_Holophagae_2	1.3	3.0	0.1	1.1	2.4	0.9	1.0	1.4	0.1	0.3	0.3	3.2	1.8
	Unclassified_Holophagae_1	2.2	3.9	0.4	2.0	2.2	2.6	0.9	1.9	0.9	0.0	1.0	1.0	1.4
	Unclassified_Chloroplast	8.0	8.0	4.4	6.1	5.8	5.2	0.3	9.7	3.2	5.0	5.5	7.1	7.8
	Unclassified_Planctomycetaceae	2.8	0.6	3.9	2.1	2.7	0.2	0.8	2.8	3.1	2.0	0.4	0.3	0.6
	SUM	35.2	36.2	20.4	33.5	23.9	24.6	14.9	34.8	29.0	35.8	28.8	31.0	34.7
F	Unclassified_Ignavibacteriales	3.3	2.1	2.6	1.3	2.4	1.8	0.0	0.0	0.7	0.7	1.6	3.7	4.2
	Unclassified_Bacteroidete	4.6	0.6	3.7	3.9	1.3	4.5	1.2	3.6	5.1	3.5	2.3	0.7	2.0
	SUM	7.9	2.6	6.3	5.2	3.7	6.3	1.2	3.7	5.7	4.3	3.9	4.4	6.2
MR	Unclassified_Acidobacteriaceae	1.9	3.0	1.6	3.1	3.9	3.6	1.8	0.7	1.1	3.9	0.8	0.4	1.8
	Bradyrhizobium	1.2	0.5	1.7	2.8	0.6	1.5	0.2	1.1	4.6	0.1	0.5	1.4	1.9
	Unclassified_Aminicenantes	4.8	5.8	2.2	5.0	0.4	3.0	3.2	3.2	3.2	3.4	4.7	1.5	4.0
	Unclassified_Rhodospirillales_1	4.6	6.3	0.6	7.6	4.6	2.6	2.4	1.5	7.9	3.7	1.7	0.6	4.1
	SUM	12.5	15.5	6.0	18.5	9.5	10.7	7.6	6.5	16.7	11.1	7.6	3.8	11.8
MRF	Unclassified_Phycisphaeraceae	4.1	3.5	6.3	3.2	0.7	7.2	2.2	6.2	3.5	6.1	8.9	5.5	5.2
	Nocardioides	4.2	3.4	3.1	2.4	0.6	5.3	2.7	4.9	2.1	5.9	2.0	2.7	4.5
	Marmoricola	4.2	3.5	0.9	3.3	2.6	2.0	2.7	0.4	3.0	3.8	1.9	2.5	0.5
	Unclassified_GOUTA4	2.2	1.8	1.4	2.3	1.0	2.4	1.3	1.9	1.2	3.5	0.1	0.6	0.9
	Tetrasphaera	2.7	4.2	0.1	4.6	4.0	0.3	0.7	0.9	5.5	4.8	0.6	3.0	1.6
	Unclassified_Actinobacteria	1.4	0.8	0.5	2.8	0.9	0.7	1.0	2.8	3.6	4.2	0.0	0.9	0.3
	Unclassified_Acidobacteria	2.1	4.2	5.9	4.3	0.8	1.9	1.8	2.1	3.9	4.4	3.4	3.3	3.4
	Unclassified_Intrasporangiaceae	5.1	5.2	3.4	4.9	6.3	3.2	1.2	0.6	5.1	4.3	1.2	4.0	2.5
	Unclassified_Rhodospirillales_2	1.1	0.8	1.6	3.6	0.7	1.0	1.3	3.6	2.9	1.8	0.2	0.7	1.6
	Unclassified_Sphingobacteriales	0.9	0.1	2.5	1.3	0.8	3.7	0.6	1.7	1.7	1.2	2.9	1.0	0.4
	SUM	28.0	27.5	25.7	32.8	18.3	27.5	15.4	25.0	32.3	40.0	21.1	24.3	21.1

"SUM" means the sum importance of all the bacterial indicator taxa enriched in each treatment as drivers for each soil nutrient cycling index.

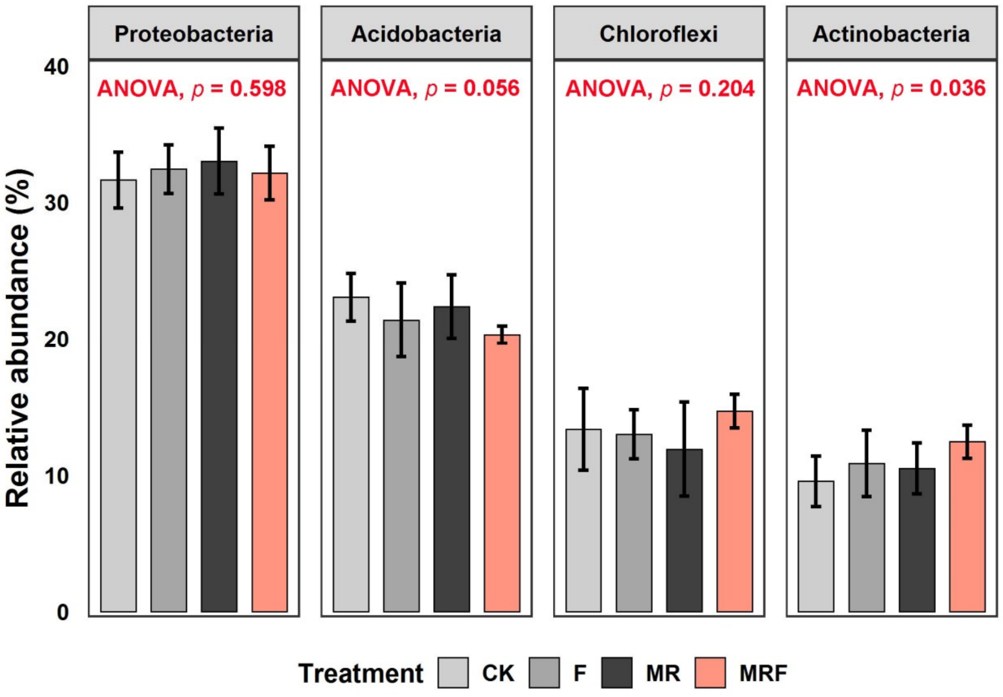

Figure S1. Relative abundances of dominant bacteria among different fertilization regimes. The error bars indicate standard deviations of means.

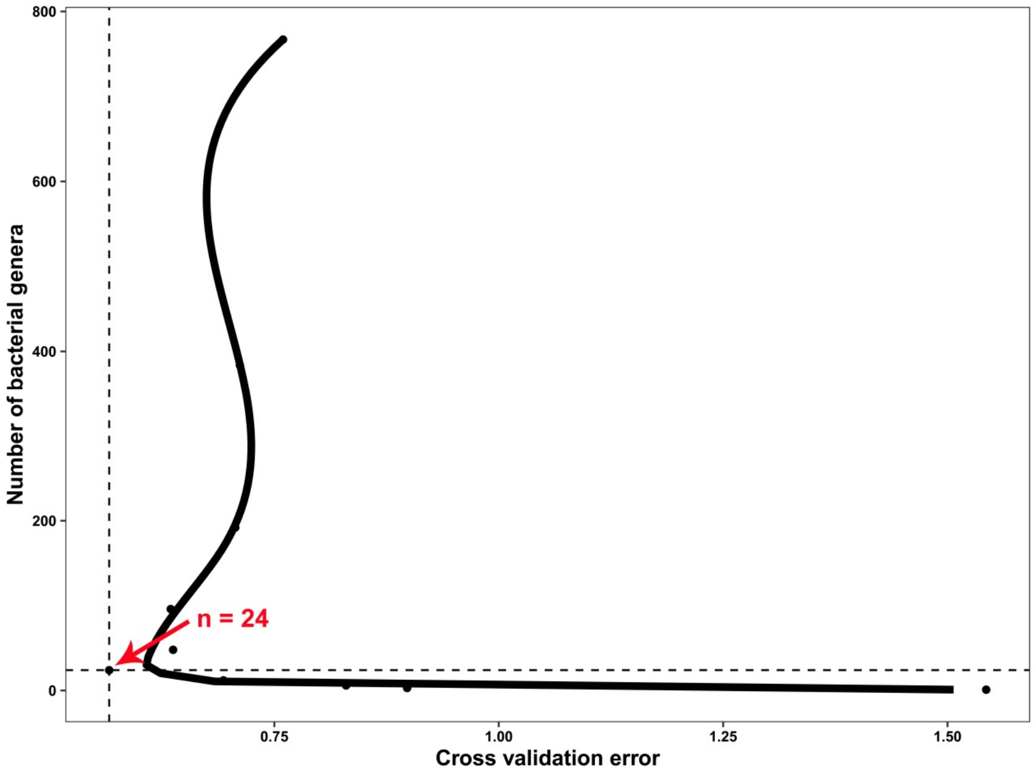

Figure S2. The number of bacterial genera against the cross-validation error curve.

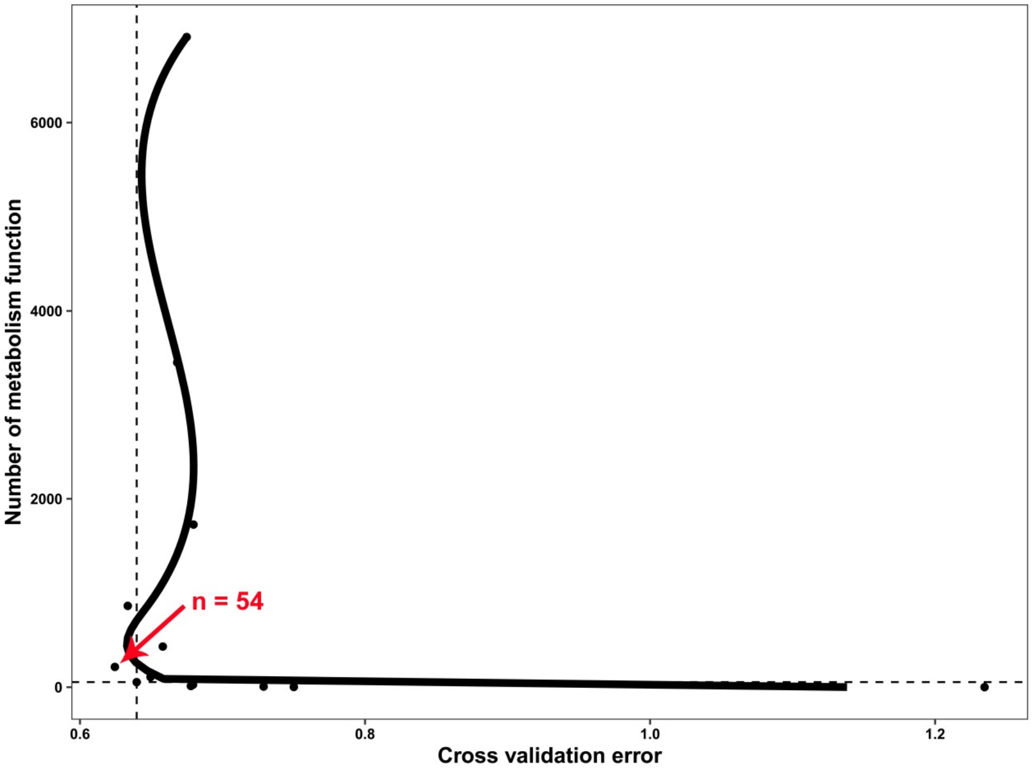

Figure S3. The number of metabolism function against the cross-validation error curve.
